# Trends in Cardiovascular Disease Mortality in US Women Veterans vs Civilians

**DOI:** 10.1001/jamanetworkopen.2023.40242

**Published:** 2023-10-30

**Authors:** Ramin Ebrahimi, Elizabeth M. Yano, Carlos A. Alvarez, Paul A. Dennis, A. Laurie Shroyer, Jean C. Beckham, Jennifer A. Sumner

**Affiliations:** 1 Department of Medicine, University of California, Los Angeles; 2 Department of Medicine, Veterans Affairs Greater Los Angeles Healthcare System, Los Angeles, California; 3 Veterans Affairs Health Services Research & Development Center for the Study of Healthcare Innovation, Implementation and Policy, Veterans Affairs Greater Los Angeles Healthcare System, Los Angeles, California; 4 Department of Health Policy and Management, University of California Los Angeles; 5 Department of Pharmacy Practice, Texas Tech University Health Science Center, Dallas; 6 Department of Research, Veterans Affairs North Texas Health Care System, Dallas; 7 Department of Population Health Sciences, Duke University School of Medicine, Durham, North Carolina; 8 Durham Veterans Affairs Medical Center, Durham, North Carolina; 9 Department of Surgery, Renaissance School of Medicine, Stony Brook University, Stony Brook, New York; 10 Northport Veterans Affairs Medical Center, Northport, New York; 11 Department of Psychiatry and Behavioral Sciences, Duke University School of Medicine, Durham, North Carolina; 12 Department of Psychology, University of California, Los Angeles

## Abstract

**Importance:**

Cardiovascular disease (CVD) remains the leading cause of death in the US. Women veterans have higher rates of CVD compared with civilian US women; however, analyses of recent trends in mortality from cardiac disease for women veterans are lacking.

**Objective:**

To investigate trends in cardiac disease mortality among women veterans over approximately the past 2 decades and compare rates with those for civilian women.

**Design, Setting, and Participants:**

In this retrospective longitudinal cohort study, US Veterans Health Administration (VHA) electronic health record data, linked with the National Death Index, were analyzed for CVD trends and rates of cardiac disease mortality among women veterans (aged 18 years or older) with VHA health care encounters from January 1, 2000, to December 31, 2017. These data were compared with a national cohort of civilian women (aged 15 years or older) in the Centers for Disease Control and Prevention Wide-Ranging Online Data for Epidemiologic Research (CDC WONDER) database, which provides cause-of-death data using death certificates for all US residents. The data analysis was performed between March 10, 2021, and November 28, 2022.

**Exposure:**

Cardiac disease mortality among women veterans and civilian women.

**Main Outcomes and Measures:**

Cardiac disease mortality was based on *International Classification of Diseases, Tenth Revision* diagnostic codes (I00-I09, I11, I13, and I20-I51 as defined by CDC WONDER). For women veterans and civilian women, crude and age-adjusted cardiac disease mortality rates (per 100 000 life-years) and 95% CIs were calculated, with the 2000 US general population as the reference for age-adjusted rates.

**Results:**

From 2000 to 2017, 817 912 women veterans engaged with VHA health care (mean [SD] age, 45.7 [17.1] years), and 19 022 cardiac disease deaths were identified (22.4% of total deaths). The crude and age-adjusted cardiac disease mortality rates, respectively, per 100 000 life-years were 200.2 (95% CI, 181.0-221.0) and 197.6 (95% CI, 175.2-222.0) in 2000 and 196.0 (95% CI, 186.1-206.4) and 208.1 (95% CI, 196.4-220.4) in 2017, reflecting stable crude rates and a 5.3% increase in age-adjusted rates. For civilian women, the crude and age-adjusted rates decreased over time from 320.7 (95% CI, 319.7-321.8) and 268.1 (95% CI, 267.3-269.0) in 2000 to 220.9 (95% CI, 220.1-221.7) and 164.7 (95% CI, 164.1-165.3) in 2017.

**Conclusions and Relevance:**

In this cohort study comparing women veterans and civilian women, cardiac disease mortality rates for women veterans did not exhibit the improvements seen for civilian women during the nearly 2-decade study period. Further research and actionable clinical interventions are warranted to improve cardiovascular care for women veterans, who represent the fastest growing group of patients within the VHA health care system.

## Key Points

**Question:**

What are the trends for cardiac disease mortality for women veterans, and how do they compare with those for civilian women?

**Findings:**

In this cohort study of 817 912 women veterans, the crude cardiac disease mortality rate from 2000 to 2017 remained flat, and the age-adjusted rate increased by 5.3%, while the corresponding rates for civilian women decreased by 31.1% and 38.6%, respectively. In the last year of the study (2017), the age-adjusted rate was 26.4% higher for women veterans compared with civilian women.

**Meaning:**

These findings suggest a lack of improvement in cardiac mortality for women veterans vs civilian women over nearly 2 decades, indicating a need for research and actionable clinical interventions to improve cardiovascular care for women veterans.

## Teaser

This cohort study investigates trends in cardiac disease mortality among women veterans over 2 decades and compares rates with those for civilian women.

## Introduction

Cardiovascular disease (CVD)—the leading cause of death in the US^1^—remains understudied, underdiagnosed, and undertreated in women.^2,3,4^ Women veterans are the fastest growing users of the US Veterans Health Administration (VHA), nearly tripling from 2000 to 2015.^5^ Approximately 10% of the 20 million US veterans are women; this number is estimated to exceed 2.2 million over the next 2 decades.^6^ As highlighted in a recent call to action,^7^ women veterans face unique cardiovascular risks and have higher rates of CVD compared with civilian US women or men veterans.^7^ This study (1) investigated trends in rates of cardiac disease mortality among women veterans using the VHA over a nearly 2-decade period and (2) compared rates to the corresponding cardiac disease mortality trends for civilian women.

## Methods

### Study Design, Setting, and Participants

In this retrospective, longitudinal cohort study, we used national VHA electronic health record data, including outpatient, inpatient, and purchased care (ie, services received from external clinicians but paid for by the VHA), from the VHA Corporate Data Warehouse^8^ to identify a cohort of women veterans aged 18 years or older who used VHA health care from January 1, 2000, to December 31, 2017. Consistent with prior work, we defined VHA health care utilization as having at least 1 inpatient or 2 outpatient clinical encounters during the study period.^9^ Race and ethnicity were self-reported in the electronic health record and were collected as part of the data collected for the original study as opposed to this subanalysis. Women who did not pass data quality control checks (eg, invalid death date, nonveterans) were excluded. As described later, we compared these women veterans with civilian women in the general US population.

This research was approved by the institutional review boards of the University of California, Los Angeles and Greater Los Angeles Veterans Affairs Healthcare System. Because of the retrospective data analysis and that many of the women studied had died, informed consent was not possible. This study followed the Strengthening the Reporting of Observational Studies in Epidemiology (STROBE) reporting guideline.

### Outcome

Our outcome was cardiac disease mortality from 2000 to 2017. Vital status and cause of death among women veterans were obtained by linking the US National Death Index database to the VHA cohort. Data for civilian women were from the Centers for Disease Control and Prevention (CDC) Wide-Ranging Online Data for Epidemiologic Research (WONDER) online database, which defines cardiac disease mortality based on *International Classification of Diseases, Tenth Revision* (*ICD-10*) diagnostic codes (I00-I09, I11, I13, and I20-I51). Any earlier *ICD* revision codes were converted to *ICD-10* diagnostic codes. The same *ICD-10* codes were used to define cardiac disease mortality for both women veterans and civilian women.

### Statistical Analysis

The data analysis was performed between March 10, 2021, and November 28, 2022. Crude and age-adjusted cardiac disease mortality rates (per 100 000 life-years) and 95% CIs were calculated for each year of the study for women veterans and civilian women. For age-adjusted cardiac disease rates, we used the 2000 US general population as the reference, as this is the reference used by CDC WONDER for generated age-adjusted death rates. Thus, to support comparison of the age-adjusted rates across women veterans and civilian women, we used the 2000 US population as the reference for both sets of analyses. The CDC WONDER data include cause of death for all ages, and they allow for age range evaluations based on 10-year categories (eg, 15-24, 25-34, and 35-44 years). However, the cohort of women veterans included those aged 18 years or older, because the earliest age for military participation is 18 years. As a result, the first age category for women veterans was 18 to 24 years vs 15 to 24 years for civilian women. However, subsequent age groups were identical. The data analysis was performed using SAS, version 9.4 software (SAS Institute, Inc).

## Results

During the study period, 833 784 women veterans were identified. Of these, 4787 did not pass data quality control and were excluded, yielding 828 997 women veterans. The number of women veterans using VHA health care grew from 197 260 in 2000 to 739 703 in 2017. During this period, 817 912 women veterans had at least 1 inpatient or 2 outpatient VHA clinical encounters and formed the women veterans analytic sample. The mean (SD) age at first VHA encounter for the analytic sample was 45.7 (17.1) years. The sample was relatively diverse with respect to race: 23.7% Black, 53.0% White, 3.2% other races [Asian, American Indian, Alaska Native, Native Hawaiian, or Pacific Islander], and 20.1% unknown race. The majority of the analytic sample was not of Hispanic ethnicity (77.7%); 6.2% of women veterans identified as Hispanic or Latina, and 16.1% had unknown ethnicity. From 2000 to 2017, 84 897 women veterans died, with 19 022 (22.4%) identified as dying of cardiac disease.

Table 1 presents crude and age-adjusted cardiac disease mortality rates for women veterans during the study period. The crude and age-adjusted cardiac disease mortality rates, respectively, per 100 000 life-years fluctuated from 243.8 (95% CI, 228.3-260.1) and 241.4 (95% CI, 225.2-258.5) in 2004 (highest) to 189.6 (95% CI, 178.8-200.9) and 172.3 (95% CI, 162.0-187.3) in 2011 (lowest). Trends from 2000 to 2017 represented relatively stable crude rates and a 5.3% increase in age-adjusted rates. Crude and age-adjusted cardiac disease mortality rates, respectively, per 100 000 life-years were 200.2 (95% CI, 181.0-221.0) and 197.6 (95% CI, 175.2-222.0) in 2000 and 196.0 (95% CI, 186.1-206.4) and 208.1 (95% CI, 196.4-220.4) in 2017.

**Table 1.** Crude and Age-Adjusted Cardiac Disease Mortality Rates in US Women Veterans, 2000-2017

Year	Total No. of women veterans	No. of deaths	Cardiac disease mortality rate per 100 000 life-years (95% CI)	
			Crude	Age adjusted^a^
2000	197 260	395	200.2 (181.0-221.0)	197.6 (175.2-222.0)
2001	252 845	573	226.6 (208.4-246.0)	218.1 (198.0-239.6)
2002	297 731	715	240.2 (222.9-258.4)	230.8 (212.1-250.6)
2003	339 017	808	238.3 (222.2-255.4)	236.7 (218.3-254.0)
2004	376 901	919	243.8 (228.3-260.1)	241.4 (225.2-258.5)
2005	410 656	986	240.1 (225.4-255.6)	237.8 (222.5-253.9)
2006	442 670	955	215.7 (202.3-229.9)	211.9 (198.1-226.4)
2007	473935	996	210.2 (197.3-223.6)	202.8 (189.6-216.6)
2008	506 518	1066	210.5 (198.0-223.5)	196.3 (183.4-209.9)
2009	539 795	1129	209.2 (197.1-221.7)	201.2 (187.4-216.6)
2010	574 265	1194	207.9 (196.3-220.1)	184.3 (171.4-197.9)
2011	605 480	1148	189.6 (178.8-200.9)	174.3 (162.0-187.3)
2012	636 425	1267	199.1 (188.3-210.4)	185.4 (173.2-198.3)
2013	664 100	1303	196.2 (185.7-207.2)	192.8 (180.6-205.6)
2014	689 308	1337	194.0 (183.7-204.7)	199.4 (187.1-212.2)
2015	710 732	1391	195.7 (185.6-206.3)	209.0 (196.6-221.8)
2016	728 208	1390	190.9 (181.0-201.2)	210.0 (197.8-222.8)
2017	739 703	1450	196.0 (186.1-206.4)	208.1 (196.4-220.4)

^a^ For age-adjusted rates, the 2000 US general population was the reference.

Table 2 presents crude and age-adjusted cardiac disease mortality rates from 2000 to 2017 for civilian women. During this period, the number of women aged 15 years or older rose from 113 969 175 to 135 458 688, with 5 644 402 cardiac disease deaths. Trends from 2000 to 2017 represented a 31.1% reduction in crude rates and a 38.6% reduction in age-adjusted rates. Crude and age-adjusted cardiac disease mortality rates, respectively, per 100 000 life-years were 320.7 (95% CI, 319.7-321.8) and 268.1 (95% CI, 267.3-269.0) in 2000 and 220.9 (95% CI, 220.1-221.7) and 164.7 (95% CI, 164.1-165.3) in 2017. The age-adjusted cardiac disease mortality rate during the final year of the study (2017) was 26.4% higher for women veterans compared with civilian women. The Figure shows the crude and age-adjusted rates of cardiac disease mortality between women veterans and civilian women.

**Table 2.** Crude and Age-Adjusted Cardiac Disease Mortality Rates in US Civilian Women, 2000-2017

Year	Total No. of women	No. of deaths	Cardiac disease mortality rate per 100 000 life-years (95% CI)	
			Crude	Age adjusted^a^
2000	113 969 175	365 520	320.7 (319.7-321.8)	268.1 (267.3-269.0)
2001	115 570 255	360 554	312.0 (311.0-313.0)	261.1 (260.2-261.9)
2002	116 825 827	355 576	304.4 (303.4-305.4)	254.7 (253.8-255.5)
2003	118 073 587	348 588	295.2 (294.2-296.2)	246.2 (245.4-247.1)
2004	119.354.958	330 100	276.6 (275.6-277.5)	230.7 (229.9-231.5)
2005	120 755 434	328 886	272.4 (271.4-273.3)	225.7 (224.9-226.4)
2006	122 164 519	315 576	258.3 (257.4-259.2)	212.6 (211.8-213.3)
2007	123 515 319	305 854	247.6 (246.7-248.5)	202.2 (201.4-202.9)
2008	124 832 870	305 247	244.5 (243.7-245.4)	198.2 (197.4-198.9)
2009	126 098 170	291 829	231.4 (230.6-232.3)	186.3 (185.6-187.0)
2010	127 025 926	290 006	228.3 (227.5-229.1)	182.2 (181.5-182.8)
2011	128 370 709	287 838	224.2 (223.4-225.0)	176.4 (175.7-177.0)
2012	129 520 417	286 917	221.5 (220.7-222.3)	172.3 (171.7-173.0)
2013	130 602 029	289 467	221.6 (220.8-222.4)	170.7 (170.1-171.3)
2014	132 037 990	288 956	218.8 (218.0-219.6)	167.6 (167.0-168.2)
2015	133 329 857	298 520	223.9 (223.1-224.7)	169.9 (169.3-170.5)
2016	134 209 029	295 694	220.3 (219.5-221.1)	165.8 (165.1-166.4)
2017	135 458 688	299 273	220.9 (220.1-221.7)	164.7 (164.1-165.3)

^a^ For age-adjusted rates, the 2000 US general population was the reference.

**Figure.**
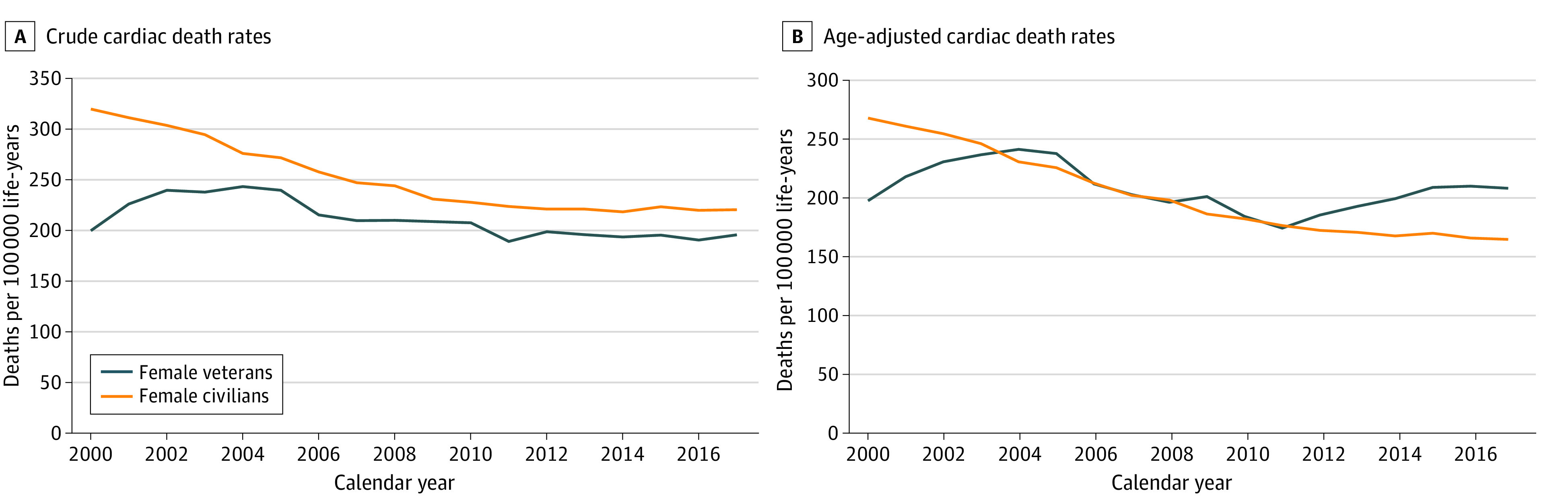
Crude and Age-Adjusted Cardiac Disease Mortality Rates for US Women Veterans and Civilian Women

Given large differences in cardiac disease mortality rates for women veterans and civilian women during the initial years of the study, and to allow for possible late adoption of updated guidelines for treatment of CVD risk factors during that period, we also compared cardiac disease mortality rates for women veterans and civilian women only during the last decade of the study period (2008-2017). Using 2008 rather than 2000 as the reference, findings were generally consistent, with a 6.9% decrease in crude rates and a 6.0% increase in age-adjusted rates for women veterans compared with a 9.7% decrease in crude rates and 16.9% decrease in age-adjusted rates for civilian women.

## Discussion

Although women veterans have higher rates of CVD than civilian women, analyses of trends in cardiac disease mortality for women veterans have been generally lacking. Such investigations are key, as women veterans are a unique and growing population. Indeed, although women have participated in the US military since the Revolutionary War,^10^ they have been increasingly represented in the military and in a wide range of roles.^11^ Moreover, in the past 2 decades, the sociodemographic profile of women veterans has changed significantly. Whereas the age of women veterans represented a bimodal distribution in 2000, with peaks in midlife (45 years) and later life (77 years), the distribution shifted in 2019 to be trimodal, with peaks at ages 35, late 50s to early 60s, and 95 years.^5^ Furthermore, in the VHA, women veterans are more diverse in terms of race and ethnicity compared with their men veteran counterparts.^5^

In this study, we documented cardiac disease mortality rates for women veterans from 2000 to 2017 and compared results with those for civilian women over the same period. Whereas civilian women demonstrated declines in crude and age-adjusted cardiac disease mortality rates over nearly 2 decades, rates in women veterans remained stable or increased slightly during the same period. Furthermore, in the final year of the study (2017), the age-adjusted cardiac disease mortality rate was 26.4% higher for women veterans than for civilian women.

Results of prior studies comparing cardiovascular mortality between women veterans and civilian women are conflicting. In postmenopausal women in the Women’s Health Initiative who were followed up for approximately 15 years (3719 women veterans), pre–Vietnam generation women veterans had higher cardiovascular mortality rates compared with civilian women; however, no differences were observed for Vietnam and later generation women veterans compared with civilian women.^12^ In addition, in nearly 10 000 women veterans who did and did not serve in Vietnam, women veterans had fewer deaths due to circulatory disease (which includes cardiac disease mortality) compared with civilian women over a 16- to 17-year follow-up.^13^ In contrast to our study of more than 800 000 women veterans engaging with VHA health care from 2000 to 2017, these studies focused on women veterans who served predominantly in Vietnam and earlier periods and used composite outcomes that included other causes of death besides cardiac disease, such as hypertension, stroke, or other circulatory or vascular conditions. While commonly used, such composite outcomes may not necessarily reflect the outcome of each subcomponent. Our goal for this analysis was to specifically investigate trends in cardiac disease mortality, as defined by the CDC, as opposed to a broader composite end point.

Using this large and relatively contemporary women veterans cohort, we found that women veterans had no clinically significant improvement in crude or age-adjusted rates of cardiac disease mortality over the study period. In contrast, cardiac disease mortality rates for civilian women decreased significantly over the same period. Alarmingly, this lack of reduction in cardiac disease mortality rate in women veterans occurred despite changes in national guidelines that lowered the threshold for identification and treatment of CVD risk factors, such as hypertension, hyperlipidemia, and diabetes.^14,15,16^ Potential explanations for the lack of improvement in cardiac disease mortality rates in women veterans may be multifactorial, including (1) increasing rates of tobacco use, (2) high treatment nonadherence rates, (3) higher prevalence of cardiovascular risk factors that exceed rates for civilian women, and (4) greater overall clinical complexity in women veterans.^7,17^ Furthermore, prevalence of nontraditional CVD risk factors, such as posttraumatic stress disorder, is higher in women veterans as opposed to civilian women,^7^ and posttraumatic stress disorder has been shown to be an independent CVD risk factor in women veterans.^9^ Additionally, specific barriers to provision of care for women veterans within the VHA are well documented^18^ and may limit engagement with preventive health care. Other factors may include financial, educational, racial, and ethnic disparities between women veterans and civilian women. Understanding factors underlying the discrepancies observed between women veterans and civilian women is an important future direction, as it can help to guide prevention and intervention efforts.

### Limitations

Similar to all retrospective database analyses, our study has limitations, including selection bias, possible misclassification, issues with representativeness, and missing data. In addition, the US population of civilian women from CDC WONDER includes women veterans who did not receive their care from VHA, although these women may be a very small percentage of the civilian women population considered. However, including these women veterans as civilian women might attenuate some differences in cardiac disease mortality rates between the 2 cohorts. We also analyzed data for women veterans who used VHA health care, defined as having at least 1 inpatient or 2 outpatient clinical encounters during the study period. Unlike some prior studies of women veterans,^10^ we did not have access to women veterans not using VHA health care, and future research is needed to determine whether those women might have different patterns of cardiac disease mortality over time. Nevertheless, with our definition of VHA health care utilization, we aimed to encompass a wide array of women veterans engaging with the VHA and not just women veterans with underlying health issues who required many encounters or those who would be most likely to experience cardiac disease mortality. Another limitation is that the youngest age group differed for women veterans (18-24 years) and civilian women (15-24 years) due to the 18-year minimum age requirement to enlist in the military. However, age-adjusted rates also revealed discordance in cardiac disease mortality trends between women veterans and civilian women. Furthermore, the choice of the standard population can influence the age-adjusted rates. Although we used the same reference population as CDC WONDER to support comparability across women veteran and civilian women analyses, research using other references is needed to determine the robustness of these findings. It would also be interesting to compare the women veteran and civilian women cohorts on various characteristics, such as cardiovascular risk factors or whether mean age changed over time according to veteran vs civilian status. However, these data could not be obtained from the CDC WONDER database, thereby precluding such comparisons. Even with these limitations, to our knowledge, this is the only study to investigate cardiac disease mortality rates over the past 2 decades in a large sample of women veterans and to include a general US population for comparison. Furthermore, our data reflect a more contemporary period as opposed to more remote periods in prior work.

## Conclusions

The sizeable reductions in cardiac disease mortality rates for civilian women over the past 2 decades are laudable, but, as this study’s findings suggest, parallel improvements were lacking in women veterans who use the VHA for health care. These results reinforce previous calls to action to prioritize research and actionable clinical interventions to improve cardiovascular care for women veterans.^7,19^

## Notes

Supplement.
Data Sharing Statement

## References

[1] Tsao CW, Aday AW, Almarzooq ZI, . Heart disease and stroke statistics-2022 update: a report from the American Heart Association. Circulation. 2022;145(8):e153-e639. doi:10.1161/CIR.0000000000001052 35078371

[2] Mehta PK, Wei J, Wenger NK. Ischemic heart disease in women: a focus on risk factors. Trends Cardiovasc Med. 2015;25(2):140-151. doi:10.1016/j.tcm.2014.10.005 25453985 PMC4336825

[3] Mehta PK, Bess C, Elias-Smale S, . Gender in cardiovascular medicine: chest pain and coronary artery disease. Eur Heart J. 2019;40(47):3819-3826. doi:10.1093/eurheartj/ehz784 31713592 PMC7963141

[4] Mauvais-Jarvis F, Bairey Merz N, Barnes PJ, . Sex and gender: modifiers of health, disease, and medicine. Lancet. 2020;396(10250):565-582. doi:10.1016/S0140-6736(20)31561-0 32828189 PMC7440877

[5] Frayne SM, Phibbs CS, Saechao F, ; Women’s Health Evaluation Initiative, Women’s Health Services, Veterans Health Administration, US Department of Veterans Affairs. Sourcebook: Women Veterans in the Veterans Health Administration. Volume 4: Longitudinal Trends in Sociodemographics, Utilization, Health Profile, and Geographic Distribution. US Department of Veterans Affairs; 2018.

[6] Veteran population. National Center for Veterans Analysis and Statistics, US Department of Veterans Affairs; 2017. Accessed November 5, 2021. https://www.va.gov/vetdata/veteran_population.asp

[7] Han JK, Yano EM, Watson KE, Ebrahimi R. Cardiovascular care in women veterans: a call to action. Circulation. 2019;139(8):1102-1109. doi:10.1161/CIRCULATIONAHA.118.037748 30779640

[8] Fihn SD, Francis J, Clancy C, . Insights from advanced analytics at the Veterans Health Administration. Health Aff (Millwood). 2014;33(7):1203-1211. doi:10.1377/hlthaff.2014.0054 25006147

[9] Ebrahimi R, Lynch KE, Beckham JC, . Association of posttraumatic stress disorder and incident ischemic heart disease in women veterans. JAMA Cardiol. 2021;6(6):642-651. doi:10.1001/jamacardio.2021.0227 33729463 PMC7970390

[10] Murdoch M, Bradley A, Mather SH, Klein RE, Turner CL, Yano EM. Women and war. What physicians should know. J Gen Intern Med. 2006;21(Suppl 3)(suppl 3):S5-S10. doi:10.1111/j.1525-1497.2006.00368.x 16637946 PMC1513175

[11] Street AE, Vogt D, Dutra L. A new generation of women veterans: stressors faced by women deployed to Iraq and Afghanistan. Clin Psychol Rev. 2009;29(8):685-694. doi:10.1016/j.cpr.2009.08.007 19766368

[12] Washington DL, Bird CE, LaMonte MJ, . Military generation and its relationship to mortality in women veterans in the Women’s Health Initiative. Gerontologist. 2016;56(Suppl 1)(suppl 1):S126-S137. doi:10.1093/geront/gnv669 26768386 PMC5881617

[13] Thomas TL, Kang HK, Dalager NA. Mortality among women Vietnam veterans, 1973-1987. Am J Epidemiol. 1991;134(9):973-980. doi:10.1093/oxfordjournals.aje.a116182 1951295

[14] Grundy SM, Stone NJ, Bailey AL, . 2018 AHA/ACC/AACVPR/AAPA/ABC/ACPM/ADA/AGS/APhA/ASPC/NLA/PCNA guideline on the management of blood cholesterol: a report of the American College of Cardiology/American Heart Association Task Force on Clinical Practice Guidelines. Circulation. 2019;139(25):e1082-e1143. 30586774 10.1161/CIR.0000000000000625 PMC7403606

[15] Whelton PK, Carey RM, Aronow WS, . 2017 ACC/AHA/AAPA/ABC/ACPM/AGS/APhA/ASH/ASPC/NMA/PCNA guideline for the prevention, detection, evaluation, and management of high blood pressure in adults: executive summary: a report of the American College of Cardiology/American Heart Association Task Force on Clinical Practice Guidelines. Circulation. 2018;138(17):e426-e483. 30354655 10.1161/CIR.0000000000000597

[16] American Diabetes Association. 10. Cardiovascular disease and risk management: *Standards of Medical Care in Diabetes-2021.* Diabetes Care. 2021;44(suppl 1):S125-S150. doi:10.2337/dc21-S010 33298421

[17] Creech SK, Pulverman CS, Crawford JN, . Clinical complexity in women veterans: a systematic review of the recent evidence on mental health and physical health comorbidities. Behav Med. 2021;47(1):69-87. doi:10.1080/08964289.2019.1644283 31403895

[18] Bean-Mayberry B, Moreau J, Hamilton AB, . Cardiovascular risk screening among women veterans: identifying provider and patient barriers and facilitators to develop a clinical toolkit. Womens Health Issues. 2022;32(3):284-292. doi:10.1016/j.whi.2021.12.003 35115227

[19] Jeon-Slaughter H, Chen X, Tsai S, Ramanan B, Ebrahimi R. Developing an internally validated veterans affairs women cardiovascular disease risk score using Veterans Affairs national electronic health records. J Am Heart Assoc. 2021;10(5):e019217. doi:10.1161/JAHA.120.019217 33619994 PMC8174271

