# Supplementary material for: Trends in Cardiovascular Disease Mortality in US Women Veterans vs Civilians
Source: JAMA Netw Open. 2023 Oct 30;6(10):e2340242. doi: 10.1001/jamanetworkopen.2023.40242 (PMC10616720; doi:10.1001/jamanetworkopen.2023.40242)
Supplement: Supplement. — Data Sharing Statement [file jamanetwopen-e2340242-s001.pdf]

Data Sharing Statement

Ebrahimi. Trends in Cardiovascular Disease Mortality in US Women Veterans vs Civilians. *JAMA Netw Open*. Published October 30, 2023. doi:10.1001/jamanetworkopen.2023.40242

Data
Data available: No

Additional Information
Explanation for why data not available: This study was based on data from the veteran's administration (VA) electronic medical records. The VA does not allow sharing of such data.
